# The Role of Endogenous Proteins on the Emulsification of Silicone Oils Used in Vitreoretinal Surgery

**DOI:** 10.1155/2020/2915010

**Published:** 2020-08-20

**Authors:** Irene Nepita, Rodolfo Repetto, Jan O. Pralits, Mario R. Romano, Francesca Ravera, Eva Santini, Libero Liggieri

**Affiliations:** ^1^Department of Civil, Chemical and Environmental Engineering, University of Genoa, Via Montallegro 1, 16145 Genoa, Italy; ^2^Department of Biomedical Sciences, Humanitas University, Via Rita Levi Montalcini 4, 20089 Pieve Emanuele, Milan, Italy; ^3^CNR-Institute for Condensed Matter Chemistry and Technologies for Energy, Via De Marini 6, 16149 Genoa, Italy

## Abstract

The present work is aimed at investigating the chemicophysical properties of the interface between silicone oils (SOs) used in vitreoretinal surgery and aqueous solutions, in the presence of surfactant biomolecules. Such molecules are thought to play an important role in the formation of SO emulsions in vitrectomised eyes, in which the natural vitreous body has been replaced with a SO. In particular, we have measured the interfacial tension (IT) and the interfacial dilational viscoelasticity (DV) of the interface between SO (Siluron 1000) and serum proteins (albumin and *γ*-globulins) at various concentrations in a Dulbecco alkaline buffer. The equilibrium IT value is relevant for the onset of emulsification, and the DV influences the stability of an emulsion, once formed. The study is complemented by preliminary emulsification tests. The experimental results show that, when proteins are dissolved in the aqueous solution, the rheological properties of the interface change. The IT decreases significantly for physiological protein concentrations, and the DV modulus achieves high values, even for small protein concentrations. The emulsification tests confirm that, in the presence of proteins, emulsions are stable on the time scale of months. We conclude that the measured values of IT in the presence of serum proteins are compatible with the promotion of droplet formation, which, in addition, are expected to be stable against coalescence. Adsorption of biomolecules at the interface with the SO is, therefore, likely to play an important role in the generation of an emulsion in eyes subjected to vitrectomy. These findings are relevant to identify strategies to avoid or control the formation of emulsions in eyes.

## 1. Introduction

The repair of complex retinal detachments (RDs) often requires vitrectomy techniques and the use of silicone oil (SO) tamponades [1]. The use of SOs is widely accepted for RD management, including cases with proliferative vitreoretinopathy (PVR), giant retinal tears, severe proliferative diabetic retinopathy, tractional retinal detachments, trauma, and severe perforating injuries. Moreover, indications exist for the use of SO expanded over time, to include chronic and persistent macular holes, myopic foveoschisis and colobomatous RD, optic disc pits, uveitis, acute retinal necrosis, and hypotony [1].

The efficacy of SOs in vitrectomy is limited by the inherent possibility of oil emulsion formation in the aqueous solution [2]. SO droplets generated by the emulsification process accumulate in the vitreous chamber and can be swallowed by macrophages, possibly leading to the development of epiretinal membranes, intraretinal gliosis, and vitreoretinal proliferation [2, 3]. Moreover, SO emulsion can generate an increase of the intraocular pressure (IOP), through occlusion of the trabecular meshwork, if oil droplets migrate to the anterior chamber. This can also lead to corneal decompensation, if the oil gets in direct contact with the corneal endothelium [4]. SO droplets have also been reported in the retrolaminar optic nerve [5]. At present, an emulsification-resistant SO does not exist [6] and further research is needed to identify oil properties suitable to fit the needs of vitreoretinal surgery.

In order for an emulsion to be generated in the vitreous cavity, energy has to be provided to the system. The source of energy is eye rotations that induce fluid motion in the vitreous chamber. SO properties are also important. SO bulk viscosity has been identified as a chemicophysical property that plays a key role, and it has been demonstrated that high-viscosity oils are more resistant to emulsification [7, 8]. This is because the higher the viscosity, the less intense the flow generated by eye rotations.

The properties of the interface between SO and aqueous are also relevant to understand the tendency of the oil to emulsify. The interfacial tension (IT) is proportional to the mechanical work per unit surface needed to create new liquid interface. Based on this definition, it is clear that low values of IT favour the process of emulsification, which substantially increases the interfacial area.

The IT between pure water and SO has been measured by various authors, and it has a value of ≈40 mN/m [9, 10]. Such a value is relatively large and does not justify the finding that emulsions invariably occur if the oil is left in the vitreous cavity for long enough. However, it is unlikely that the interface between aqueous solution and SO is empty of molecules that can act as surfactants. Surfactants are molecules of different types (low molecular weight molecules, polymers, and proteins) that, because of their amphiphilic nature, accumulate spontaneously at the interface and reduce the IT. Therefore, they can be efficient emulsifiers. Endogenous molecules (proteins, lipids) in the vitreous chamber, the presence of which is favoured by the postsurgery inflammatory state of ocular tissues, can adsorb at the interface, possibly modifying significantly the interfacial rheological properties [11, 12]. In this context, only few authors considered the effect of the presence of surfactant molecules on the tendency of SOs to emulsify with water, characterising various SOs in terms of their rate of emulsification [7, 13].

In the presence of surfactants, IT is a dynamic quantity, which attains an equilibrium value only for interfaces at rest. In fact, in a surfactant solution, a thermodynamic equilibrium is established between the concentration of surfactant molecules in the liquid volume and at the interface. When the interface area, *A*, changes in time with respect to its equilibrium value *A*^0^, a variation of the IT (*γ*) with respect to its initial value (*γ*^0^) occurs, which can be interpreted as a dilational stress. This can be written as the sum of two terms

(1) $$\begin{array}{c} \Delta \gamma =E\frac{\Delta A}{A^{0}}+\eta \frac{d}{dt}\left(\frac{\Delta A}{A^{0}}\right), \end{array}$$

with Δ*γ* = *γ*(*t*) − *γ*^0^ and Δ*A* = *A*(*t*) − *A*^0^. The coefficients *E* and *η* are termed dilational surface elasticity and viscosity, respectively. In the case of small amplitude harmonic perturbations of the surface, it can be shown that the viscoelastic behaviour of the interface is characterised by two quantities, *E*′ and *E*^″^, which depend on the testing frequency. *E*′ is the dilational elasticity and *E*^″^ is directly linked to the dilational viscosity [14]. Such quantities are typically arranged, for mathematical convenience, into a complex number *E* = *E*′ + *iE*′′, with $i=\sqrt{-1}$ (see Section 2.3). The complex modulus *E* measures the dilational viscoelasticity (DV) of the interface.

DV plays an important role in the stabilisation of emulsions against phase separation. After formation, an emulsion can separate again in the liquids forming it, under the effect of various processes. Among them, droplet coalescence is the most apparent, which occurs due to the rupture of the liquid film separating two droplets. In the early stage of such a process, droplets approach each other and their interface flattens in the contact area. Since the drop volume does not change, such a deformation causes an increase of the drop surface area, resulting in a dilution of the surfactant adsorbed at the interface. In turn, this causes a local increase of the IT that tends to contrast the droplet deformation and, therefore, counteracts coalescence. At the same time, after the dilution of the adsorbed layer, the concentration of surfactant at the interface is no longer at equilibrium with the one in the solution. This triggers adsorption of new surfactant molecules from the solution, which contrasts the increase of IT. These intricate dynamic processes (interface deformation versus surfactant transport) determine the capability of a surfactant to act as an efficient emulsion stabiliser, by resisting the rupture of the thin liquid film between approaching droplets.

DV provides a measure of the IT response to variations of the interfacial area in the presence of surfactants. Large values of the DV modulus imply a significant increase of the dynamic IT after droplet deformation and, therefore, a stronger counteraction to the deformation itself. This results in droplets which are less prone to coalesce and, therefore, in stable emulsions. By its nature DV is a quantity related to the dynamic processes due to the perturbation of the droplet area. Therefore, its values depend not only on the type and concentration of the surfactants but also on the specific features of the area perturbation, such as the deformation rate and amplitude [15].

The aim of the present work is to investigate experimentally the capability of biomolecules to act as surfactants, adsorbing at the water–SO interface, and to quantify their role on the equilibrium IT and the DV.

## 2. Material and Methods

### 2.1. Working Fluids

The SO used in the experiments is Siluron 1000 cs (Fluoron GmbH, Germany), which is composed of ultrapure polydimethylsiloxane and is of common use in ophthalmic surgery. Ultrapure water utilised for the study was obtained by a Milli-Rho/Milli-Q integrated system (Millipore). We have first characterised the interfacial properties between SO and ultrapure water. These measurements are used as a benchmark reference for comparison with existing results in the literature and also to verify the effect of adding endogenous molecules.

We then studied the effect of endogenous proteins on the interfacial properties between an aqueous solution and SO. Two proteins were selected for the experimental activity: bovine serum albumin (Sigma, A2153-50G) and *γ*-globulins from bovine blood (Sigma G7516-10G). These proteins were chosen as they are present in highest concentration in blood serum [16]. Using bovine proteins allowed us to easily obtain them in suitable amounts. These proteins have been shown to be excellent models of human ones, as far as the study of interfacial properties is concerned [17–19]. Solutions of these molecules were prepared in a Dulbecco phosphate-buffered saline (DPBS, Sigma D8662), simulating the in vivo protein environment. Protein concentrations ranged between 0% (pure buffer) and 100% of the corresponding physiological protein concentration in blood serum. In particular, we considered 50 g/L a physiological concentration in the case of albumin and 25 g/L for *γ*-globulins [20, 21]. These solutions were then brought to 35°C, immediately before performing the measurements.

In order to avoid contamination from surface active molecules, all glass and plastic wares and pieces of equipment brought in contact with the fluids were first carefully cleaned with standard procedures adopted in surface science laboratories. Between different cycles of measurements, the absence of contamination was checked by measuring the IT of ultrapure water, verifying that at 20°C, it assumed a value close to 72.5 mN/m, stable over several minutes.

### 2.2. Measurements of the IT

The IT was measured adopting the Drop Shape Technique (DST), using the tensiometer PAT1 (Sinterface Technology, Berlin). The technique relies on the acquisition, through a digital camera and an algorithm ensuring a resolution below 2 *μ*m, of a pendant drop profile. The shape of the drop under the action of gravity depends on the IT and on the density difference between the two fluids. Once the densities of the two fluids are known, the IT can be obtained by a best fitting procedure of the theoretical drop profile [22] to the acquired one. For the present application, we formed a drop of the aqueous solution in a SO bath in the DST cell and took measurements at 35°C, in order to simulate physiological conditions.

DST is applicable to liquids with an appreciable density difference; in fact, if the two liquids were isodense, the drop would take a spherical shape and the IT result would be undetermined. Since in our case the density difference between SO and aqueous solution is relatively small, very accurate measurements of the densities are required for the method to be reliable. For SO, density measurements as a function of temperature have been kindly provided by Rutherford Appleton Laboratory in Oxfordshire, UK (Dr. Mario Campana), and have been performed employing the densimeter Anton Paar DMA4100 M, which has a precision of 0.1 kg/m^3^. At 35°C, we found a value of SO density of 961.2 kg/m^3^.

For the densities of protein aqueous solutions, we first considered values calculated as weighted averages between the density of the DPBS (1007.5 kg/m^3^, corresponding to the value of a 10 kg/m^3^ solution of NaCl at 35°C) [23] and the density of the blood serum at 35°C (1025 kg/m^3^) [24]. The densities of single protein aqueous solutions were then calculated assuming that the density of the solution of albumin plus *γ*-globulins in physiological concentration equals that of serum.

### 2.3. Measurements of the DV

DV values were measured using the oscillating drop method, based on the measurement of the IT response to harmonic oscillations of the interfacial area [25, 26]. The interfacial area of the drop, *A*, is forced to oscillate sinusoidally, with frequency *f* and amplitude $\tilde{A}$, around a reference value *A*^0^

(2) $$\begin{array}{c} A-A^{0}=\Delta A=\tilde{A}\sin\left(2\pi ft\right). \end{array}$$

The method is based on the assumption that, at all times, the shape of the drop is in equilibrium with the instantaneous value of the IT, which can, therefore, be measured as in the static case. The IT, however, can vary in time in response to the perturbation of the area, owing to surfactant adsorption/desorption processes.

For small amplitude perturbations, the IT response is linear; thus, the value of *γ* also oscillates harmonically in time with frequency *f*, so that

(3) $$\begin{array}{c} \gamma -\gamma^{0}=\Delta \gamma =\tilde{\gamma }\sin\left(2\pi ft+\phi \right), \end{array}$$

where *γ* is the actual value of the IT and *γ*^0^ is its equilibrium reference value. The amplitude of IT variations, $\tilde{\gamma }$, and the phase shift, *ϕ*, between the interfacial area and the IT response are related to the equilibrium and kinetic characteristics of the adsorption process.

In this case, the complex DV is recast as

(4) $$\begin{array}{c} E=\frac{\Delta \gamma }{\Delta A/A^{0}}=\frac{\tilde{\gamma }}{\tilde{A}/A^{0}}e^{i\phi }. \end{array}$$

Because of its large bulk viscosity, SO opposes a significant resistance to the expansion/contraction of the drop volume at high frequencies of oscillations. This might result in deviations of the drop shape from that at mechanical equilibrium. This effect can be limited by reducing as much as possible the amplitude of the perturbation.

The estimate of the phase angle, *ϕ*, typically results in larger errors than that of the amplitude $\tilde{\gamma }$. For this reason, we focused our analysis on the modulus of the DV, defined as $|E|=\tilde{\gamma }/\left(\tilde{A}/A^{0}\right)$.

### 2.4. Description of a Typical Test

A typical experiment starts with the formation of a fresh (nearly free from adsorbed surfactant) interface of aqueous solution in SO. This is obtained by abruptly stopping the quick formation and detachment of a train of drops at the tip of a needle. The experiment then proceeds in two phases. Right after the drop is formed, its surface area is kept constant until the IT reaches the equilibrium value. This is a standard test to follow the kinetic of adsorption at liquid interfaces. In the presence of surfactants, during this phase, a decrease of IT from an initial value, corresponding to the IT of the newly formed interface, to the final equilibrium value is observed. In the absence of surfactant molecules, as we expect in the case of the SO-ultrapure water interface, the IT remains constant and equal to its initial value.

In the second phase of the test, following the previous one without interruption, the DV was measured. The drop area was varied sinusoidally in time, with an oscillation amplitude $\tilde{A}=0.02\cdot A^{0}$ and for a set of different frequencies, in the range 0.001 Hz to 0.1 Hz. Higher frequencies were not tested, since the effect of SO bulk viscosity would have induced unacceptable distortions in the IT response (nonharmonic in time). For each frequency, a number of oscillation periods were applied, from 3 at the lowest frequency to 10 for the largest one.

**Figure 1:**
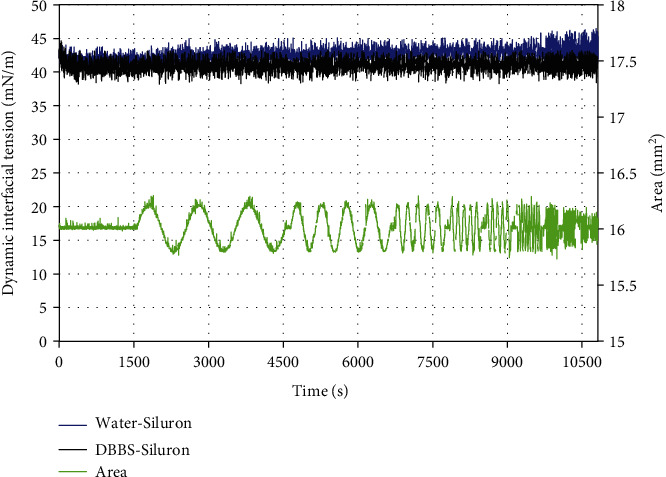
Time evolution of the dynamic IT of the SO-DPBS (black) and SO-water (blue) interfaces (left *y*-axis). For the first 1800 s, the interfacial area is kept fixed. From there on, oscillations of the interfacial area with an amplitude of 2% are imposed, for a range of different frequencies. The green line represents the interfacial area variation in time (right *y*-axis).

In Figure 1, the green curve shows the measured surface area of the drop in a typical experiment. From the acquired data, for each frequency, the amplitudes and phases of the IT response and of the drop area signals were obtained with a Fourier analysis and DV values were calculated according to Equation (4).

### 2.5. Emulsification Tests

Emulsions have been produced according to the double-syringe method [27]. We used two 5 mL syringes connected by a 15 mm long Luer-lock connector, with an inner diameter of 1 mm. Each syringe was initially filled with one of the two liquids to be emulsified. The liquids were then alternatively and completely exchanged from one syringe to the other by operating the system manually. Each stroke lasted about two seconds, and we applied five cycles. In this way, emulsion forms due to shearing forces. The syringes were then stored in vertical position to assess the time evolution of the emulsion.

## 3. Results

In Figure 1, we show the results obtained in the absence of proteins, for the interface between SO and both water and DPBS. For the SO-water interface, the measured IT is close to 42 mN/m, which is consistent with reported measurements [9]. Both systems show a small and relatively quick decrease of the IT right after the formation of the drop. However, when the unsteady phase of the experiment starts, the IT does not show any response to surface area variation, which indicates the absence of adsorption processes at the interface. Within the experimental error, we find that the IT for the interface between SO and DPBS has the same value as for the interface between SO and pure water. We then conclude that in both systems, the content of surface active impurities is negligible.

**Figure 2:**
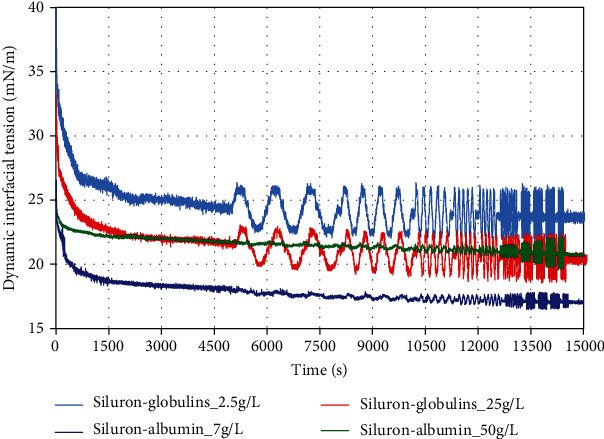
Time evolution of the dynamic IT between SO and buffer solutions containing *γ*-globulins or albumin. The curves refer to different concentrations, in which values are normalised with the corresponding value in the blood. 0.1 light blue, 1.0 red, 0.14 blue, and 1.0 green.

In Figure 2, we show the corresponding results obtained adding into the DPBS either albumin or *γ*-globulins. For each protein, we report the data corresponding to 1 and ≈1/10 of its average physiological concentration in the blood. Let us consider *γ*-globulins first (light blue and red curves in the figure). For the smallest value of concentration (light blue) at the beginning of the experiment, IT values significantly differ from those observed in the absence of proteins and they decrease on a time scale of thousands of seconds, nearly achieving an equilibrium value of ≈23 mN/m, which is almost half of the value observed in the absence of proteins. This confirms the occurrence of an adsorption process of the protein at the interface. The oscillatory phase of the experiment starts at 5000 s. When surface oscillations are imposed, the IT also shows large variations, indicating the existence of a significant DV. For the physiological value of *γ*-globulins concentration (red curve), the results are similar but the IT decreases to an even smaller value: about 21 mN/m.

For albumin (Figure 2, blue and green curves), the adsorption process is faster and the IT equilibrium values are smaller than for *γ*-globulins, achieving values of ≈17 mN/m  with a normalised concentration of 0.14. In addition, the amplitude of IT oscillations in response to variations of the surface area is much smaller and almost vanishes for the largest concentration value. This is because the adsorption time scale is quite fast, so that the IT recovers very rapidly its equilibrium value in response to the applied variations of the surface area.

**Figure 3:**
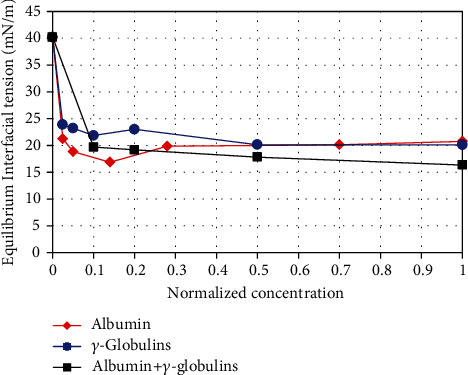
Equilibrium IT as a function of protein concentration. Concentration is normalised with the value corresponding to the concentration in blood.

Figure 3 shows the equilibrium IT for solutions containing albumin, *γ*-globulins, and a mixture of the two proteins, in physiological ratio. The concentrations are normalised with their average physiological content in blood. Equilibrium values are extrapolated from the adsorption kinetics experiments, applying a long-time approximation scheme, as proposed by Makievski et al. [28]. In all cases, the IT decreases as the concentration of surfactant molecules increases, down to a saturation value. Such a value for both proteins is achieved already at a concentration of about 1/10 of their physiological value, with IT reduction of ≈50% with respect to the SO-DPBS interface. The effect of the mixture of the two proteins in physiological ratio does not differ from the effects of the single proteins, which calls for a poor interaction between them, once adsorbed.

**Figure 4:**
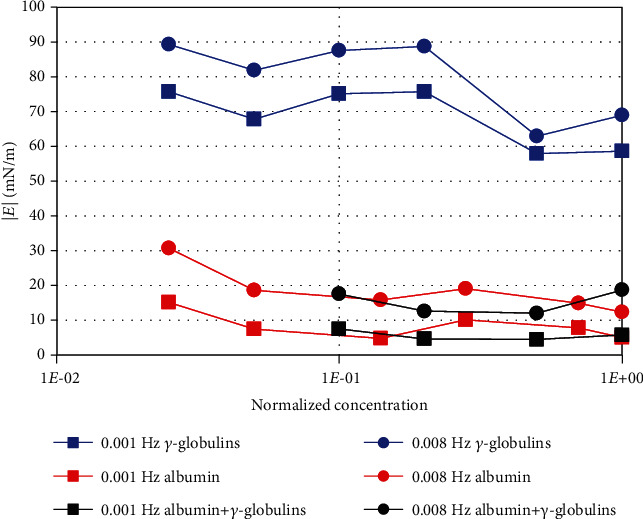
DV modulus as a function of protein concentration for different perturbation frequencies.

The DV modulus is shown in Figure 4 as a function of the normalised concentration and for two different frequencies, among those measured. In agreement with what discussed previously (Figure 2), the DV modulus is larger for *γ*-globulins than for albumin solutions. We note that the values measured for *γ*-globulins are much larger (even more than an order of magnitude) than those observed for ordinary surfactants. The values measured for the mixture of proteins are similar to those obtained for albumin, which therefore appears to play a dominant role on the interface rheological behavior.

**Figure 5:**
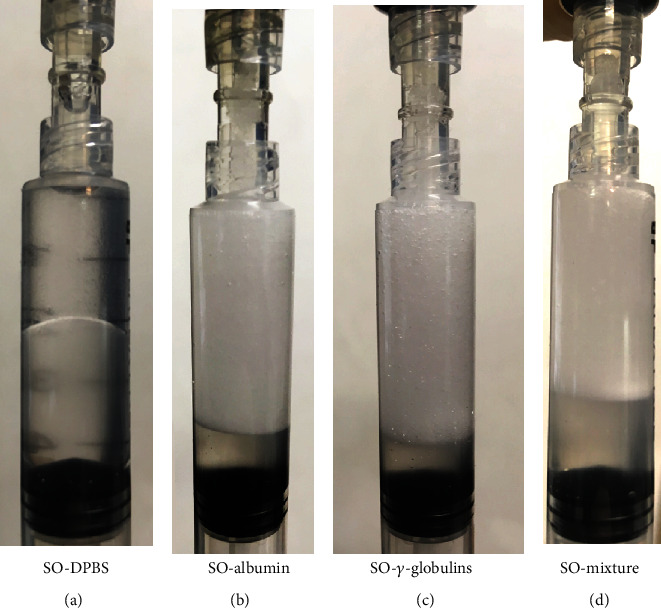
1 : 1 volume ratio emulsions between SO and aqueous phases, 1 hour after formation. (a) DBPS; (b) albumin 0.1 normalised concentration in DBPS; (c) *γ*-globulins 0.1 normalised concentration in DBPS; (d) albumin+*γ*-globulins 0.1 normalised concentration in DBPS.

The capability of blood proteins to stabilise SO emulsions was tested with albumin, *γ*-globulins, and albumin+*γ*-globulins, assuming a value of the normalised concentration equal to 0.1, that is, 10% of the physiological content in the blood. As shown in Figure 5, after one hour from formation, the emulsion produced with the bare buffer is fully separated, while all those containing the proteins are stable. Keeping these emulsions in quiet conditions at room temperature, the situation did not change significantly even after ninety days, when a consistent volume of emulsion was still observed. For *γ*-globulins solutions, these observations are comparable with those of Nakamura et al. [12], though the authors used a different emulsification method.

## 4. Discussion

In the present work, we have investigated the role of endogenous surfactant molecules on the interfacial properties between SO and aqueous solutions. In particular, we have considered endogenous biomolecules (albumin and *γ*-globulins) that can be produced by inflammation of ocular tissues in response to surgery [13, 29]. We have studied whether such proteins can modify the interfacial properties enough to make them compatible with the generation of an emulsion. This is a typical complication in vitrectomised eyes, in which SOs have been injected as tamponades.

We have focused on two properties: the interfacial tension (IT) and the dilational interfacial viscoelasticity (DV). The former quantity is associated with the possible generation of an emulsion: low values of IT promote the generation of emulsions.

On the other hand, large values of the DV modulus confer high mechanical stability to the liquid interface, preventing droplet coalescence once the emulsion is formed and determining, therefore, its evolution in time [30, 31].

We have first performed experiments with a saline buffer solution and measured values of IT similar to those reported in the literature that almost invariably refer to the interface with pure water [9, 10].

We have then performed experiments with aqueous solutions containing blood proteins (*γ*-globulins and albumin). These measurements show that, even at small protein concentrations, the interfacial rheological properties change significantly and the equilibrium IT decreases to ≈20 mN/m, which is approximately half of its original value (interface between SO and bare buffer) and is compatible with the formation of an emulsion [32]. We have also found that both in the presence of the single proteins and of their mixtures, large values of the DV modulus are attained, even 3 to 10 times larger than for ordinary surfactants. This indicates that, once formed, emulsions may be quite stable in time. It is thus confirmed that even a small concentration of proteins leads to relevant surface active effects.

The capability of albumin, *γ*-globulins, and their mixture to form emulsions stable over the time scale of months was preliminary confirmed by comparative emulsification tests. The emulsions have been formed using a very simple and standard method, which consists in exchanging the fluids between two syringes. In this way, the emulsion is induced by shear forces. This approach has allowed us to demonstrate the appearance of very stable emulsions, even for relatively low concentrations of blood proteins. In reality, it is well accepted that SO emulsification is induced by mechanical energy, mostly introduced in the system by eye rotations. In this context, some studies have evaluated the role of eye movements adopting realistic domains. Chan et al. [33] performed emulsification tests in a model of the vitreous chamber made of poly (methyl-methacrylate) and found that the addition of high-molecular components to a base oil affected the viscosity of the tamponade fluid and thus its propensity to emulsify. Additional emulsification experiments have been carried out in order to investigate the role of different SO densities [34] and the effect of encircling scleral buckle [8] on SO flow within the vitreous chamber. All these studies, however, did not take into account the role of organic material in the aqueous solution. For the above reasons, more extensive tests would be useful to investigate emulsification under realistic conditions and assess the role of protein concentration.

## 5. Conclusions

The presence of endogenous proteins in the aqueous solution in contact with the SO induces a significant decrease of the IT and is responsible for relatively large values of the DV. This scenario is compatible with the formation and persistence of a SO emulsion. Surgeons need to be aware that, in the presence of endogenous proteins produced by the surgery itself or specific ocular conditions (trauma, young age,), significantly less mechanical energy is necessary to break SO interface into small droplets, while the resulting droplets remain stable against coalescence. Thus, the incidence of SO emulsion in the postoperative period increases. These findings are relevant to identify strategies to avoid or control the formation of emulsions in the eyes.

Even if important effects of representative blood proteins are evidenced by the present study, the interfacial properties and the emulsification under realistic vitrectomised eyes can be quite different. In fact, owing to the presence of further blood components that may act in addition or synergy with the investigated proteins, we expect even more important effects. These aspects will be addressed by future studies using whole human blood serum.

## Data Availability

The data set containing the values behind the means, standard deviations, and other measures reported in the manuscript and the values used to build graphs are available from the corresponding author upon request.
